# A multicentre phase II randomised trial of weekly docetaxel/gemcitabine followed by erlotinib on progression, *vs* the reverse sequence, in elderly patients with advanced non small-cell lung cancer selected with a comprehensive geriatric assessment (the GFPC 0504 study)

**DOI:** 10.1038/bjc.2011.331

**Published:** 2011-09-20

**Authors:** H LeCaer, F Barlesi, R Corre, H Jullian, S Bota, L Falchero, A Vergnenegre, C Dujon, J Y Delhoume, C Chouaid, the GFPC 0504 Team

**Affiliations:** 1 Services de Pneumologie, CH Route Montferrat 83300, Draguignan, France; 2 Service d'Oncologie Multidisciplinaire & Innovations Thérapeutiques et Centre d'Investigations Cliniques, Université de la Méditerranée, APHM, Marseille, France; 3 Services de Pneumologie, CHU, Rennes, France; 4 Services de Pneumologie, Martrigues, France; 5 Services de Pneumologie, CHU, Rouen, France; 6 Services de Pneumologie, Villefranche, France; 7 Services de Pneumologie, CHU, Limoges, France; 8 Services de Pneumologie, Le Chesnay, France; 9 Services de Pneumologie, Perigueux, France; 10 Services de Pneumologie, Saint Antoine, APHP, UMPC, Paris, France

**Keywords:** elderly, co-morbidities, phase II trial, chemotherapy, lung cancer

## Abstract

**Background:**

Elderly cancer patients form a heterogeneous population in which therapeutic decision-making is often difficult. The aim of this randomised phase II trial was to evaluate the feasibility and activity of weekly docetaxel/gemcitabine (DG) followed by erlotinib after progression (arm A) *vs* erlotinib followed by DG after progression (arm B) in fit elderly patients with advanced non small-cell lung cancer (NSCLC).

**Methods:**

Elderly chemotherapy-naive patients with stage IIIB/IV NSCLC were selected after a comprehensive geriatric assessment (socioeconomic, cognitive, depression, ADL and IADL assessments). The primary endpoint was the time to second progression (TTP2). Overall survival (OS), the time to first progression (TTP1) and safety were secondary endpoints.

**Results:**

Between July 2006 and November 2008, 22 centres enrolled 100 patients. TTP2 was 7.5 and 5.8 months in arm A and arm B, respectively; TTP1 was 4.7 and 2.7 months; and the median OS time was 9.4 and 7.1 months; the respective 1-year survival rates were 36.2 and 31.4%. There was no major unexpected toxicity.

**Conclusion:**

These results suggest that weekly DG, followed by erlotinib, is a promising treatment for fit elderly patients with NSCLC; the efficacy of the reverse sequence was insufficient to recommend it for EGFR-non-selected patients.

Between 30 and 40% of non small-cell lung cancer (NSCLC) cases are diagnosed in patients over 70 years of age, raising specific issues of age, comorbidity and toxicity (Pallis *et al*, 2010). Most elderly patients are either undertreated or receive non-validated schedules (Jatoi *et al*, 2005; Pallis *et al*, 2010). They are largely underrepresented in therapeutic trials, and little clinical research takes their specificities into account (Pallis *et al*, 2010). Yet, the value of specific studies of elderly subjects has been clearly demonstrated (Jatoi *et al*, 2005).

The recommended first-line treatment for patients under 65 with metastatic NSCLC and good performance status (PS) consists of dual-agent platinum-based chemotherapy. There is no consensus on the management of elderly NSCLC patients, although adapted platinum-based chemotherapy seems feasible in high-selected elderly subjects (Pfister *et al*, 2004; Gridelli *et al*, 2007b; Quoix *et al*, 2010; Felip *et al*, 2011). Although the ELVIS trial (ELVIS Group, 1999), single-agent chemotherapy has been the rule in this setting. However, dual-agent therapy without a platinum salt seems possible for patients selected on the basis of a geriatric assessment taking comorbidities into account (Weiss and Langer, 2009). Among the available non-platinum-based chemotherapy regimens, the docetaxel/gemcitabine (DG) combination is considered one of the most promising (Georgoulias *et al*, 2005; Pujol *et al*, 2005). A weekly schedule has shown good efficacy and acceptable toxicity in several phase II trials in elderly patients (Hainsworth *et al*, 2001; LeCaer *et al*, 2007; Pallis *et al*, 2008). One of our previous studies, an open-label phase II trial involving 50 elderly patients selected according to their age, Charlson score and PS, gave a 34% response rate, a median time to progression (TTP) of 5 months, and a median overall survival (OS) time of 7 months (LeCaer *et al*, 2007). Targeted therapies have given promising results in elderly populations. In the pivotal BR21 study, second-line erlotinib had the same efficacy in the subgroup of patients over 70 as in the entire population (Wheatley-Price *et al*, 2008). Targeted therapies are also a potential first-line option for elderly patients with advanced NSCLC. In an EGFR-non selected population over 70 years of age, erlotinib controlled the disease in 51% of cases, with a median survival time of 10.9 months (Jackman *et al*, 2007). Erlotinib was well tolerated, and there was a significant improvement in key symptoms (Jackman *et al*, 2007).

One difficulty in this setting is the heterogeneity of elderly populations. The use of a comorbidity score and a comprehensive geriatric assessment (CGA) can help to identify fragile patients and to define a more homogenous group of fit elderly patients (Pal *et al*, 2010).

In view of these reports, we used a CGA to select a population of fit elderly patients for a multicentre, randomised phase II study of the feasibility and activity of weekly DG followed by erlotinib after progression (arm A), *vs* the reverse sequence (arm B).

## Patients and methods

### Study design

This was a multicentre, open-label, phase II study (GFPC 0504). As we wished to evaluate all the active treatment periods, the primary endpoint was the time to TTP2, as determined with the RECIST method (Therasse *et al*, 2000); the secondary endpoints were OS, TTP1, the objective response rate (complete+partial responses), the disease control rate (objective responses+stable disease), safety, and quality of life (QoL). The protocol was approved by an independent ethics committee in Marseille (French institutional ethic review board), on behalf of all participating centres, and the study complied with Good Clinical Practices and the Helsinki Declaration. The trial had been registered under NCT number 00418704.

### Eligibility criteria

The geriatric inclusion criteria combined age, the Charlson score (Charlson *et al*, 1987), comorbidity and PS and geriatric items, to select a population of fit elderly patients (Table 1). The geriatric non-inclusion criteria were age >89 years and a combined comorbidity-PS score or CGA score incompatible with the values shown in Table 1.

We applied the following oncologic inclusion criteria: cytologically or histologically proven NSCLC of stage IV or IIIB with T4 stage by neoplastic pleural effusion, according to the TNM Classification of Malignant Tumours, 6th edition (Sobin and Wittekind, 2002), not previously treated with chemotherapy, a measurable tumour, life expectancy more than 3 months, and biological status compatible with chemotherapy (bilirubin <1.25 ULN, transaminase activity <3 ULN, alkaline phosphatase <2.5 ULN, polymorphonuclear neutrophil count >1.5 G l^−1^, and platelet count >100 G l^−1^). The oncologic non-inclusion criteria were histological status (small-cell lung cancer, bronchioloalveolar carcinoma), prior chemotherapy, symptomatic brain metastases, unstable heart disease, uncontrolled infection, grade >2 neuropathy, a history of metastatic malignancy in the last 5 years and permanent contraindications to the use of steroids.

Treatment arm A consisted of a maximum of three 8-week treatment cycles with weekly docetaxel 30 mg m^−2^ for 6 consecutive weeks and gemcitabin 900 mg m^−2^ on weeks 1, 2, 4 and 5, followed by a 2-week treatment-free period; CT assessments were done after each chemotherapy cycle (8 weeks) and, in case of non progression after three cycles, every 8 weeks. Patients who progressed were treated with erlotinib (150 mg per day) and assessed every 8 weeks. In arm B, patients received erlotinib first (150 mg per day), with an assessment every 8 weeks; patients who progressed received the first-line chemotherapy schedule used in arm A.

Patients in both arms systematically received epoietin beta (30 000 units once a week) when the haemoglobin level fell below 11 g dl^−1^. Neutrophil growth factors consisted of curative lenograstim for febrile neutropenia, or secondary lenograstim prophylaxis from D3 to D5. Chemotherapy administration could be postponed for up to 2 weeks, if the patient had not fully recovered from the haematological toxicity of the previous cycle, with a 25% dose reduction. Specimen collection for determining EGFR status was not part of the initial study design.

### Efficacy

Objective tumour responses were assessed at the end of each 8-week chemotherapy cycle, every 8 weeks during erlotinib therapy, and every 8 weeks in patients who did not progress after chemotherapy. Second progression-free survival was calculated from the date of randomisation to the date of disease progression (after the second line of treatment if the patients receive two lines, after the first-line if the patient progressed and did not receive a second line) or death of any cause, or the last on-trial tumour assessment. OS was calculated from the date of randomisation to the date of death from any cause, or the last date the patient was known to be alive. Patients would be considered as assessable if they received at least 8 weeks of erlotinib therapy or one cycle of chemotherapy (8-week period). All responses were centrally reviewed and confirmed by a panel of experts convened by GFPC (Groupe Français de Pneumo-Cancérologie).

Patients were monitored for adverse events, biological abnormalities, vital signs and electrocardiographic changes using NCI-CTC version 2.0.

### Statistical analysis

In this one-step phase II trial, we assumed that the median TTP2 was 6 months for the strategy with chemotherapy first (arm A), and 8 months for the strategy with erlotinib first (arm B). A sample size of 47 patients per group would have an 80% power with a type I error of 5% to detect differences between the two arms, based on the log-rank test. This number was rounded up to 50 patients per arm to take into account losses to follow-up and ineligibility. The analysis will be performed on an intention-to-treat basis.

Quantitative data were expressed as the population, number, mean, s.d. and range; qualitative data were expressed as the population, number and frequency. All tests were two-sided, and significance was assumed at *P*<0.05. Quantitative variables were compared with Student's *t*-test or with Wilcoxon's test when the groups were too small or the data were not normally distributed. Qualitative parameters were compared with the *χ*^2^-test for theoretical group sizes above five, and with Fisher's test in other cases. PFS and OS were assessed by means of Kaplan–Meier analysis. Statistical analyses were done with SAS software version 8.02 (Institute INC, Cary, NC, USA). QoL was assessed during the initial work-up (intention-to-treat) and at the end of each 8-week cycle for patients receiving chemotherapy, and every 8 weeks during erlotinib treatment, using the Spitzer index (Spitzer *et al*, 1981) and the Lung Cancer Symptom Scale (LCSS; Hollen *et al*, 1994). Each item of the Spitzer score is attributed a score of 0 to 2, with higher values reflecting a better health. A mean global score is then calculated. Each item of the LCSS questionnaire is scored from 0 to 10; the higher the score, the more intense the symptom. The LCSS questionnaire yields two scores: a symptom score calculated from the first six items (appetite, fatigue, cough, breathlessness, haemoptysis, and pain), and a global score derived from the last three items (symptom severity, discomfort during routine activities, and QoL).

Role of the sponsors: the sponsors had no role in the study design, study realisation, data analysis or manuscript preparation. GFPC have the result property. The data were analysed by the GFPC statistician and interpreted by the authors.

## Results

Between July 2006 and November 2008, 22 centres enrolled 100 patients in this study (Table 2). The patients in the two arms were not significantly different; mean age was respectively 76 and 75.7 years in arms A and B, with respectively 21 and 16% of patients over 80 years old. Three patients under 70 years were enrolled and one of them was subsequently excluded (ineligible). No significant difference was noted in the Charlson score, comorbidities, or the geriatric assessment at baseline (Table 3). The CGA allowed us to select a population of fit elderly patients, with a mean MMSE of 29.7, only moderate malnutrition, independence in the ADL and IADL scores, and a high global score (EGS *K*=18/20 on average) in both arms (Table 3).

**Figure 1:**
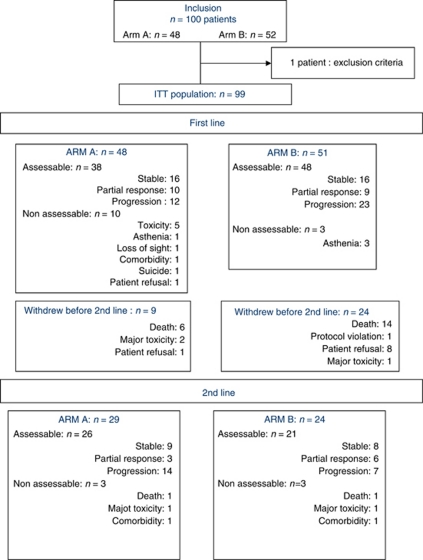
Flow chart of included patients; arm A: DG followed by erlotinib if progression, arm B: erlotinib followed by DG if progression (assessable patient: at least 8 weeks of treatment).

All the patients in arm A received at least one dose of DG chemotherapy and 38/48 (79%) patients were assessable after first-line chemotherapy; 60.4% of patients received a second line of treatment with erlotinib and 89% of them were assessable (Figure 1). In arm B, all the patients received at least one dose of erlotinib and 94% were assessable; 47% received second-line chemotherapy and 87.5% of them were assessable (Figure 1). Among the patients who could be assessed after their first line of treatment, significantly more patients in arm A than in arm B received a second line of treatment (76% *vs* 50%, *P*=0.013). In arm A, the mean number of first-line chemotherapy cycles per patient was 1.83 and the mean duration of second-line erlotinib treatment 4.7 months. In arm B, the mean duration of first-line erlotinib treatment was 3.1 months and the mean number of second-line chemotherapy cycles per patient was 1.83. The mean relative dose intensity of gemcitabin was 79% and 74% in arm A (first line) and arm B (second line), respectively. For docetaxel, the rates were 85% in arm A (first line) and 90% in arm B (second line).

**Figure 2:**
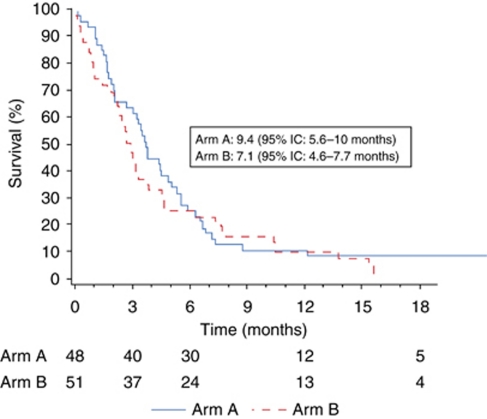
Time to second progression (months); arm A: DG followed by erlotinib if progression, arm B: erlotinib followed by DG if progression.

**Figure 3:**
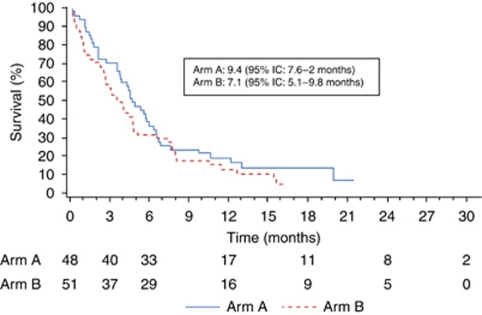
Overall survival (months); arm A: DG followed by erlotinib if progression, arm B: erlotinib followed by DG if progression.

The first objective of the study was not met, as there was no significant difference between the two arms in terms of TTP2 (7.5 and 5.8 months, respectively, in arms A and B, *P*=0.53; Figure 2). TTP1 was 4.7 and 2.7 months (*P*=0.53); median OS was 9.4 and 7.1 months (*P*=0.26, Figure 3); and the 1-year survival rate was 36.2 and 31.4%. Central review showed no differences in objective responses or disease control (Table 4). There were no significant differences with the results of the investigators' assessments (data not shown). The factors predictive of survival were PS (*P*=0.03), CGA nutritional status (*P*=0.04), and pain (*P*=0.01). PS was predictive of TTP2 (*P*=0.005).

**Figure 4:**
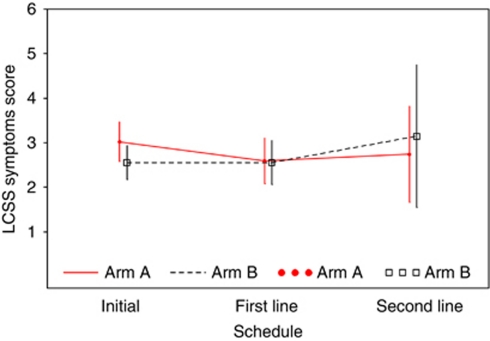
QoL – LCSS: score changes according to symptoms (*n*=99); mean and confidence interval of symptom score at baseline, and after first- and second-line treatment; arm A: DG followed by erlotinib if progression, arm B: erlotinib followed by DG if progression.

Safety was assessable for all the patients. The most common grade 3–4 adverse events were asthenia in both the arms, neutropenia and thrombopenia with DG, and cutaneous reactions with erlotinib (Table 5). Only 6.3% of patients in arm A developed grade 3–4 anaemia, probably because of the routine use of epoietin beta. Around 75% of the patients (39 in arm A, 37 in arm B) completed the QoL assessment before treatment; the median global LCSS score, the median symptom score and the global Spitzer score were similar in the two arms and showed little deterioration of QoL after treatment (Figure 4). In arms A and B, 29 and 27 patients, respectively, completed the QoL assessment after 8 weeks, and 11 and 8 patients, respectively, after 16 weeks. These scores did not change significantly during treatment, even when the response to treatment was taken into account (Figure 4).

## Discussion

In this phase II randomised trial in fit elderly patients with advanced NSCLC, selected with a CGA, the TTP2 was 7.5 months with weekly DG followed by erlotinib, and 5.8 months with erlotinib followed by DG; the respective median times to TTP1 were 4.7 and 2.7 months and the median OS was 9.4 and 7.1 months, respectively. The definition of ‘elderly’ is controversial. The epidemiological literature uses age 65 years to define elderly patients, but 70 years is also commonly used. This trial, designed in 2005, used an age of 65 years, although 70 years is now a more common cut-off. Only three patients aged between 65 and 70 years, one of whom was ineligible, were included in this study.

The first originality of this study is that the patients were selected on the basis of geriatric criteria combining age, PS and comorbidity, but also, in keeping with SIOG recommendations (Extermann *et al*, 2005), functional, mental, social and nutritional status and daily activities. The main advantage of these evaluations is to improve the stratification of elderly patients and thereby to allow valid comparisons across different studies. The Charlson and comorbidity scales, even if they do not correlate with PS, are an essential complement to the CGA (Repetto *et al*, 2002). To validate treatments tested in clinical trials, and to make the results of different studies comparable, it seems relevant to use a full geriatric assessment such as CGA, allowing fit patients to be separated from the vulnerable and fragile, pending prospective validation of geriatric screening tools (Luciani *et al*, 2010; Pal *et al*, 2010; Soubeyrand *et al*, 2011).

The second originality of this study is that the second-line treatment was fixed in each arm, allowing us to evaluate the performance of each treatment sequence.

Our results for first-line DG chemotherapy in elderly patients are in line with those of previous studies by our team and others. Thus, the same DG combination used in a open phase II study of 50 patients selected on the basis of the PS and Charlson score, without a CGA, yielded median PFS and OS times of 5 and 7 months, respectively (LeCaer *et al*, 2007). In a retrospective analysis of 192 NSCLC patients, at least 70 years of age, who received first-line DG chemotherapy, the overall response rate was 30.2%, the median TTP1 was 4.5 months and the median OS was 9.2 months (Pallis *et al*, 2008). A phase III study compared the efficacy of weekly docetaxel and the DG combination; the median OS times were similar (5.1 and 5.5 months, respectively), but the median TTP1 was significantly longer in the patients who received DG (4.8 months *vs* 2.9 months; *P*=0.004). Both regimens were generally well tolerated (Hainsworth *et al*, 2007). Sequential treatment with gemcitabine followed by weekly docetaxel gave similar results, with median TTP1 and OS times of 4.8 and 8.0 months, respectively (Tibaldi *et al*, 2008). Recent studies of non-platinum-containing doublets have given disappointing results. A bi-weekly combination of pemetrexed and gemcitabine Blakely *et al*, 2009) gave a median PFS of 3.5 months in 45 elderly patients and caused grade 3/4 neutropenia in 22% of patients. A randomised phase II trial (Gridelli *et al*, 2007a) comparing pemetrexed monotherapy (500 mg m^−2^) with pemetrexed followed by gemcitabine gave a very poor median OS of around 5 months in both arms. In contrast, there was no major toxicity. A more recent phase III trial involving patients over 70 years old showed the superiority of the carboplatin–taxol combination over navelbine ou gemcitabine monotherapy (Quoix *et al*, 2010). These results need to be confirmed, especially in terms of tolerability.

Targeted therapies are a potential option for elderly patients with advanced NSCLC. Jackman *et al* (2007)tested first-line erlotinib in a phase II study with 80 NSCLC patients over 70 years of age. Erlotinib was well tolerated, with an encouraging response rate of 10% and disease stabilisation in 41% of cases. There was a significant improvement in key symptoms (dyspnoea, cough, fatigue and pain) and the median OS was 10.9 months. These results are far better than those obtained here, in terms of both OS and TTP1, but Jackman *et al*'s (2007) population included more women and more patients with adenocarcinoma. In contrast, the same percentage of patients received a second line of treatment. Gefitinib was compared with oral vinorelbine in a phase II randomised trial involving a very similar population (predominantly female elderly patients, most with adenocarcinoma). The median times to first progression were 2.7 and 2.9 months, respectively, with median OS times of 5.9 and 8 months (Crinò *et al*, 2008). Only 19% of patients in the gefitinib arm received a second-line treatment, compared with 29% of patients in the vinorelbine arm. There were fewer treatment-related grade 3–5 adverse events with gefitinib (12.8%) than with vinorelbine (41.7%). In this study, a substantially lower percentage of first-line erlotinib-treated patients received second-line chemotherapy. Most patients had a PS of 2 and more, and could not receive chemotherapy, even a non-platin doublet. This difference may have had a role in the inferior overall results of this treatment approach.

In the second-line setting, a retrospective analysis of the BR.21 trial examined the influence of age on erlotinib outcomes (Wheatley-Price *et al*, 2008). There was no significant age-related difference in PFS or OS in the erlotinib or placebo arm. However, compared with young patients, elderly patients had significantly more overall and severe toxicity (grade 3–4; 35% *vs* 18% *P*<0.001), were more likely to discontinue treatment as a result of treatment-related toxicity (12% *vs* 3% *P*<0.0001), and had a lower relative dose intensity (64% *vs* 82% received >90% of the planned dose; *P*<0.001). The toxicity of erlotinib in our CGA-selected population was acceptable and was not associated with a high rate of treatment withdrawals.

If age alone is not a contraindication to treatment in elderly subjects, another promising approach in this population is to use, in addition to the CGA, genetic selection criteria (Provencio *et al*, 2009). Customised cisplatin treatment, based on a reduction in nucleotide excision repair function, is one attractive approach, whereas mitotic checkpoint gene status can be used to guide docetaxel therapy. Several heritable mutations accelerate the onset of multiple aging phenotypes. The process of normal aging, with the involvement of DNA repair pathways and the impairment of mitotic checkpoint genes, could be a major way for customising treatment in elderly patients (Provencio *et al*, 2009).

In conclusion, these results suggest that weekly DG, followed by erlotinib when progression occurs, is a promising treatment for fit elderly NSCLC patients. The efficacy of the reverse sequence was insufficient to recommend it for EGFR-non-selected patients, as recently reported (Thomas *et al*, 2011). The use of a CGA for future trials in this setting appears to be crucial (Vamvakas *et al*, 2009; Des Guetz *et al*, 2010; Quoix *et al*, 2010). A new generation of clinical trials specifically designed for elderly subjects is needed, and should include the development and validation of new measures and tools for determining biological age. We have now started a large national phase III multicentre study involving patients over 70 years of age with advanced NSCLC, in which treatment allocation will be based on a strategy using a simplified geriatric scale (SGS), followed by CGA if abnormal, by comparison with a strategy based on standard criteria (PS and age), with no specific geriatric assessment (Corre, 2010).

## Figures and Tables

**Table 1:** Geriatric inclusion criteria

**Age (Charlson)**	**Dependence for ADL and IADL**	**Geriatric syndrome^a^**	**Comorbidity**	**Charlson score**	**PS**	
			0–2	(2–4)	0–1	Ineligible^b^
	No	0	0–2	(2–4)	2	Eligible^c^
65–69 (2)			3–4	(5–6)	0–1	Eligible^c^
			3–4	(5–6)	2	Ineligible^d^
			0–1	(3–4)	0–1	Eligible^c^
70–79 (3)	No	0	0–1	(3–4)	2	Ineligible^d^
			2–5	(5–8)	0–2	Ineligible^d^
80–89 (4)	No	0	0	(4)	0–1	Eligible^c^
			1–4	(5–8)	0–1	Ineligible^d^

Abbreviations: ADL=activities of daily living; IADL=instrumental of daily living; PS=performance status.

a Geriatric syndrome: dementia, urinary or faecal incontinence, falls.

b Autonomous.

c Fit.

d Vulnerable.

**Table 2:** Characteristics of the patients; arm A: DG followed by erlotinib if progression, arm B: erlotinib followed by DG if progression

	**Arm A (*n*=48)**	**Arm B (*n*=51)**
Age, mean, (years)	76.0±4.65	75.7±4.11
Gender: male (%)	29 (60.4)	30 (58.8)
Weight loss >5% (%)	19 (36.6)	18 (36)
		
*Smoker*		
Current (%)	6 (12.8)	8 (15.7)
Former (%)	26 (54.2)	25 (49)
Never smoker (%)	15 (31)	15 (29.4)
Unknown (%)	1 (2)	3 (5.9)
		
*Performance status*		
0 (%)	22 (46.8)	21 (41.2)
1 (%)	21 (44.7)	28 (54.9)
2 (%)	4 (8.5)	2 (3.9)
		
*Stage*		
IIIB (%)	6 (12.5)	4 (7.8)
IV (%)	42 (87.5)	47 (92.2)
		
*Histology*		
Squamous cell (%)	11 (22.9)	8 (15.7)
Adenocarcinoma (%)	28 (58.3)	29 (56.5)
Undifferentied (%)	9 (18.8)	14 (27.5)
		
Charlson (mean±CI)	0.521±0.825	0.353±0.770
		
*Charlson score*		
0 (%)	29 (60.4)	38 (74.5)
1–2 (%)	17 (35.4)	11 (21.6)
>2 (%)	2 (4.2)	2 (4)
		
Comorbidities (mean) age+Charlson (range)	3.15 (2–4)	3.12 (2–4)
		
Simplified Charlson score (mean)	3.44±4.04	3.12±3.66

Abbreviations: CI=confidence interval; DG=docetaxel and gemcitabine.

**Table 3:** Comprehensive geriatric assessment; arm A: DG followed by erlotinib if progression, arm B: erlotinib followed by DG if progression

		**Arm A (*n*=48)**	**Arm B (*n*=51)**
	**Score maximum**	**Mean score**	**(Min/max)**
Socioeconomic conditions	12	11.5 (7/12)	11.4 (8/12)
Cognitive assessment	14	13.6 (10/14)	13.5 (11/14)
Emotional status and depression scale	9	0.8 (0/4)	1 (0/6)
Sensorial status	4	3.8 (3/4)	3.8 (2/4)
Nutritional risk	14	10.2 (5/14)	10.2 (5/14)
QoL Iris scale	6	5.4 (4/6)	5.2 (3/6)
ADL	6	6 (6/6)	6 (6/6)
IADL	4	4 (4/4)	4 (4/4)
Incontinence scale	4	4 (4/4)	4 (4/4)
Falls and mobility	10	9.8 (8/10)	9.9 (9/10)
Pain	32	7.9 (0/32)	5.3 (0/24)
Global score (EGSK)	20	18 (10/20)	18 (10/20)
MMS de Folstein	30	29.7 (25/30)	29.7 (24/30)

Abbreviations: ADL=activities of daily living; DG= docetaxel and gemcitabine; EGSK=name of software used for our CGA; IADL=instrumental of daily living; MMS=Mini Mental Status; QoL=quality of life.

**Table 4:** Efficacy: arm A: DG followed by erlotinib if progression, arm B: erlotinib followed by DG if progression

	**Arm A (*n*=48)**	**Arm B (*n*=51)**
Time to second progression (months)	7.5±3.6	5.8±2.2
Time to first progression (months)	4.7±2	2.7±1.5
Follow-up (median, months)	9.4±4.2	7.1±2.2
		
*Objective responses (first line)*		
Not assessable (%)	10 (20.8)	3 (5.9)
Stable (%)	16 (33.3)	16 (31.4)
Progression (%)	12 (25.0)	23 (45.1)
Partial response (%)	10 (20.8)	9 (17.6)
Complete response (%)	0 (0.0)	0 (0.0)
		
*Objective responses (second line)*		
Not assessable (%)	22 (45.8)	30 (58.8)
Stable (%)	9 (18.8)	8 (15.7)
Progression (%)	14 (29.20)	7 (13.7)
Partial response (%)	3 (6.3)	6 (11.8)
Complete response (%)	0 (0.0)	0 (0.0)

Abbreviation: DG=docetaxel and gemcitabine.

**Table 5:** Adverse events (>5% of patients); arm A: DG followed by erlotinib if progression, arm B: erlotinib followed by DG if progression

	**Arm A (*n*=48)**		**Arm B (*n*=51)**	
**First line toxicity**	**Grade 1/2**	**Grade 3/4**	**Grade 1/2**	**Grade 3/4**
*Haematologic*				
Anaemia (%)	29 (60.4)	3 (6.3)	12 (23.5)	–
Neutropenia (%)	21 (43.8)	15 (31.3)	–	–
Thrombocytopenia (%)	16 (35.4)	3 (6.3)	–	–
				
*Non haematologic*				
Cutaneous (%)	5 (10.5)	1 (2.1)	37 (72.6)	5 (9.8)
Asthenia (%)	33 (68.8)	3 (6.3)	15 (29.4)	4 (7.9)
Diarrhoea (%)	21 (43.7)	1 (2.1)	10 (19.6)	2 (4)
Constipation (%)	6 (12.5)	1 (2.1)	5 (9.8)	–
Nausea (%)	10 (20.9)	–	1 (2)	–
Vomiting (%)	11 (22.9)	–	6 (11.8)	–
Alopecia (%)	7 (14.6)	–	5 (9.8)	–
Pulmonary (%)	4 (8.4)	2 (4.2)	9 (17.6)	2 (4)
Peripheral neuropathy (%)	5 (10.4)	–	–	–
Anorexia (%)	4 (8.4%)	–	6 (11.8)	1 (2)

	**Arm A (*n*=29)**		**Arm B (*n*=24)**	
**Second line toxicity**	**Grade 1/2**	**Grade 3/4**	**Grade 1/2**	**Grade 3/4**
*Haematologic*				
Anaemia (%)	4 (13)	–	20 (83)	–
Neutropenia (%)	–	–	5 (20.8)	4 (16.6)
Thrombocytopenia (%)	2(5)	–	10 (41.6)	1 (3)
				
*Non haematologic*				
Cutaneous (%)	13 (44)	–	2 (8.3)	1 (3)
Asthenia (%)	9 (31)	3 (10)	16 (55)	3 (12)
Diarrhoea (%)	3 (10.3)	–	4 (16.6)	–
Constipation (%)	1 (3.4)	–	7 (29.1)	–
Nausea (%)	2 (5)	–	6 (25)	–
Alopecia (%)	–	–	2 (8.3)	–
Pulmonary	2 (5)	–	5 (20)	3 (12)
Peripheral neuropathy (%)	–	–	2 (8.3)	–
Anorexia (%)	–	–	3 (12)	–

Abbreviation: DG=docetaxel and gemcitabine.

## APPENDIX

### The GFPC0504 team

Professor Vergnenegre A, Professor Melloni B, CHU Limoges; Dr Professor Barlesi F, Dr Gimenez C, Dr Greillier L, CHU Marseille; Dr LeCaer H, Dr Barriere JR, CH Draguignan; Dr Gerinière L, CHU Lyon; Dr Bombaron P, CH Mulhouse; Dr Crequit J, CH Beauvais; Dr Auliac JB, CH Mantes; Dr Poirier R, Dr Le Treut J, CH Aix; Dr Berard H, HIA Toulon; Dr Thomas P, Dr Muller P, CH Gap; Dr Fournel P, CHU St Etienne; Dr Robinet G, Dr Andre M, CHU Brest; Dr Grivaux M, Dr Locher C, CH Meaux; Dr Berdah JF, Clinique Esperance Hyeres; Professor Chouaid C, Dr Baud M, CHU Paris St Antoine; Dr Bota S, CHU Rouen CN; Dr Paillotin D, CHU Rouen BG; Dr Corre R, Dr Lena H, CHU Rennes; Dr Chouabe S, CH Charleville; Dr Delhoume JY, CH Perigueux; Dr Dujon C, CH Le Chesnay; Dr Jullian H, Dr Simonian H, CH Martigues; Dr David P, CH Elbeuf; Dr Falchero L, CH Villefranche; Dr Janicot H, CHU Clermont-Ferrand; Dr Monnet I, CHI Creteil.
